# Validation of ACG Case-mix for equitable resource allocation in Swedish primary health care

**DOI:** 10.1186/1471-2458-9-347

**Published:** 2009-09-18

**Authors:** Andrzej Zielinski, Maria Kronogård, Håkan Lenhoff, Anders Halling

**Affiliations:** 1 Blekinge Centre of Competence, SE-371 81 Karlskrona, Sweden; 2 Lund University, Department of Clinical Sciences in Malmö, General Practice/Family Medicine SE-205 02 Malmö, Sweden

## Abstract

**Background:**

Adequate resource allocation is an important factor to ensure equity in health care. Previous reimbursement models have been based on age, gender and socioeconomic factors. An explanatory model based on individual need of primary health care (PHC) has not yet been used in Sweden to allocate resources. The aim of this study was to examine to what extent the ACG case-mix system could explain concurrent costs in Swedish PHC.

**Methods:**

Diagnoses were obtained from electronic PHC records of inhabitants in Blekinge County (approx. 150,000) listed with public PHC (approx. 120,000) for three consecutive years, 2004-2006. The inhabitants were then classified into six different resource utilization bands (RUB) using the ACG case-mix system. The mean costs for primary health care were calculated for each RUB and year. Using linear regression models and log-cost as dependent variable the adjusted R^2 ^was calculated in the unadjusted model (gender) and in consecutive models where age, listing with specific PHC and RUB were added. In an additional model the ACG groups were added.

**Results:**

Gender, age and listing with specific PHC explained 14.48-14.88% of the variance in individual costs for PHC. By also adding information on level of co-morbidity, as measured by the ACG case-mix system, to specific PHC the adjusted R^2 ^increased to 60.89-63.41%.

**Conclusion:**

The ACG case-mix system explains patient costs in primary care to a high degree. Age and gender are important explanatory factors, but most of the variance in concurrent patient costs was explained by the ACG case-mix system.

## Background

The main objective of health care is to improve health [1]. This is a complicated task, as the complexity of how health is created is not fully understood. One of the factors that enable the improvement of health as much as possible is to have an adequate allocation of resources. Results show that presence of functioning primary health care (PHC) is an important factor for the health of the population, but also regarding the costs of specialized care [2,3]. Consequently, the allocation of resources within the PHC is important for creating as much health as possible with limited resources.

Health care in Sweden is predominantly public with only a few private hospitals. About 13% of general practitioners work in the private sector. The Swedish government finances almost all health care and patients pay the same rates both in public- and private health care. However proportion of private PHC is currently increasing due to new laws. Patients can choose without limitations whether they want to be listed with a public- or a private PHC practice and the County Councils serves financier for all PHC.

The allocation of resources within PHC in Sweden has historically mainly been based on demographics and in some cases socioeconomic factors of the population, within each County Council [4]. In Sweden the demographics are changing and the average age of the population is rising [5]. This changes prevalence of disease in the population, as the risk of, for example, chronic diseases' increases with age. In addition, the occurrence of more than one chronic disease is a common state for many elderly patients [2]. Single diagnoses or only basic characteristics of the individuals are not enough to describe the complexity of co-morbidity and consumption of resources. A more sophisticated way to allocate resources would be to examine the individual need of health care of each citizen. One system that answers to this demand is the ACG case-mix system, developed by Johns Hopkins University [6,7]. The ACG case-mix system is based on the assumption that illness and clustering of illnesses are associated with certain individuals more than others. The classification is thus based on the presence or non-presence of illnesses and diagnoses in the individual. By this individual classification each patient is assigned to a certain level of morbidity or co-morbidity, where individuals with a certain degree of co-morbidity have a similar need for health care resources. The ACG case-mix system has been shown to explain much of the variance in the costs of PHC in different countries [8,9].

In the Swedish setting research on the ACG case-mix system has initially been demonstrated to be a useful foundation for explaining cost, but only in a small study in two (PHC) centres [10,11]. As Swedish health care generally has no information on individual patient costs, other approaches have been used. Much of the variation in polypharmacy, as a proxy for health care costs, in an elderly population was shown to be explained by the system [12]. The aim of this study was to examine to what extent the ACG case-mix system can contribute to explaining and estimating costs in Swedish PHC at an individual level in combination with age and gender.

## Methods

### Study population

The study was carried out in Blekinge County Council, with about 150,000 inhabitants, in the south-eastern part of Sweden. Data from private practices were not accessible, which reduced the number of patients by 20,000. Blekinge County Council has the responsibility for all PHC given in Blekinge. This includes both public and private providers with an agreement with the County Council. The organization in Blekinge consists of 12 different public PHC centres. Within the County Council there are also some privately managed PHCs.

To be able to perform the study, two different sources of information were combined: firstly, the costs calculated for different activities in PHC and secondly information on the patients and diagnoses from the electronic patient records. All information was obtained from Blekinge County Council, and collected during 2004, 2005 and 2006. The study was approved by the research ethics committee at Lund University.

### Dependent variable: Logarithm of concurrent primary health care costs (log-cost) calculated by patient-level costing

Individual patient costs are not automatically registered or calculated in Swedish PHC. To access this information, many of the county councils in Sweden instead retrospectively calculate the patient-level costing based on the ledger costs. PHC in Blekinge County Council has been performing these calculations since 2004, which gives information on patient-level costing [11,13]. The cost calculations are performed in co-operation with personnel from different areas of each of the included PHC centres. All costs are calculated in Swedish Crowns. Costs in our study included expenses for encounters to GPs, district nurses, physiotherapists, x-ray and laboratory tests. We excluded costs for prescribed drugs, costs for renting real estate, patients' travel expenses, psychological counselling, podiatric care, night care nurses and dementia care nurses, healthcare centres for immigrants, preventive healthcare for children, preventive care during pregnancy and counselling of adolescents in use of contraceptives.

### Independent variable: Co-morbidity

The Johns Hopkins Adjusted Clinical Groups (ACG) Case-Mix System measures co-morbidity on an individual level. The assumption made is that the pattern of diseases and diagnoses, rather than single diagnoses, better shows the level of co-morbidity and need for health care resources in PHC. The categorization of the pattern is based on five clinical dimensions: the duration, severity, diagnostic certainty, aetiology and need for specialist care of each diagnosis. The first step of the ACG case-mix system algorithm is to identify one or more adjusted diagnosis groups (ADG) for each individual, based on the registered ICD-10 codes. The ACG case-mix system allows all diseases, even newly discovered ones, to be classified according to the system and categorized into one of the specific ADGs. As each individual patient may have one or more ADGs, the combination of these groups is of major importance for which of the final ACGs the patient is assigned to. After the ADG categorization a branching algorithm supplies one specific ACG, out of nearly 100, to each patient. In the assignment of a particular ACG, when appropriate, age and gender are also included in the branching algorithm. These calculations are made for diagnosis over a specified period of time, in this study corresponding to one year. The resemblance of individuals within a specific ACG is that they have experienced a similar need for health care resources and similar morbidity pattern during the year [6].

For analytical reasons one more aggregation of the data is made. The individuals are divided, with respect to their ACG, into one out of six different Resource Utilization Bands (RUB) [14]. The need of PHC resources increases with the level of RUB. Thus RUB 0 indicates little or no need and RUB 5 very high need of PHC resources.

### Primary Health Care practice

Blekinge County Council has 12 public PHC practices. Each public PHC practice has about 10,000 or more inhabitants enrolled. In this study some of the PHC practices are combined, as they belong to the same administrative organization, with a common supervisor. This leaves ten different public PHC practices in the data sample used. The organization of health care in Blekinge focuses on the PHC as always being the first instance of contact with health care for the inhabitants, except in cases of acute and severe disease or trauma.

As a complement to the public PHC practices there are also some private PHC practices, both larger practices with several PHC physicians and smaller practices with only one PHC physician. The inhabitants listed with private PHC practices correspond to less than twenty percent of the total population in the county council. As these practices do not use the same electronic patient record system, it is not possible to include these practices or their listed patients in the study. There are also a few non-listed inhabitants who cannot be included in the study, but these correspond to less than one percent of the inhabitants.

### Demographics - Age and Gender

Unlike most of the other county councils in Sweden, Blekinge County Council has a population where the percentage of male inhabitants is greater than or similar to the percentage of females [15]. The shares of male and female inhabitants listed with the private PHC practices are similar to the shares in the public practices, although the share of males is slightly higher in the private practices.

The personal characteristics age and gender are used as complementary explanatory variables in the models examined.

### Statistical Analysis

The aim was to study to what degree level of co-morbidity, as measured by the ACG case-mix system, could explain concurrent individual patient's costs in PHC. Statistically the first step was to combine specific costs of the activities assigned to patients and registered in the electronic patient records. Due to the costs being highly positively skewed the best transformation was found to be the logarithm form of the patient costs (log-cost) which was used as the dependent variable in the analyses. Multiple linear regressions were then performed to evaluate the performance of the different models. The variance of log-cost was examined in the first model with gender as the explanatory variable for each year of interest separately. In the subsequent models age, PHC and RUB were added to the analysis consecutively. Finally, the original individual ACG categories were used in a model instead of the RUB. All explanatory variables were strictly treated as dummy variables.

To statistically evaluate the performance of the different models, the adjusted R^2 ^(coefficient of determination) was used. The adjusted R^2 ^allows the interpretation of the models in terms of how great a percentage of the variance in log-cost was explained by the different models for each separate year. Through the approach of using consecutive models and adding independent variables to these, it is possible to assess how the coefficient of determination is changed by increasing the number of independent variables. The consecutive models for each year separately were compared using the Likelihood-ratio test to ascertain the significance of adding more variables to reduce unexplained variance.

## Results

Blekinge County Council has about 150,000 inhabitants, more than eighty percent of whom could be included in this study for the years 2004, 2005 and 2006 (Table 1).

**Table 1:** Population listed at public and private PHC and non-listed inhabitants.

	**2004**		**2005**		**2006**	
**PHC**	**n**	**Proportion** **(%)**	**n**	**Proportion** **(%)**	**n**	**Proportion** **(%)**

Public	120 738	80.86	125 372	83.44	122 394	81.09
Private	27 976	18.74	24 760	16.48	28 499	18.88
Non-listed	604	0.40	121	0.08	42	0.03

Total	149 318	100.00	150 253	100.00	150 935	100.00

PHC - primary health care

Less than twenty percent of the inhabitants were excluded from the study because they were listed with private practices. The share of male individuals excluded was similar to the proportion of inhabitants enrolled at the public PHC centres for the different years (Table 2). An increase of cost was connected with a higher RUB that the individual belonged to in a concurrent model (Table 3). The mean RUB values are highly significant (p < 0.001) for all the years examined, except RUB 5. The coefficients within each RUB are similar between the years, with the greatest differences in RUB 5 (Table 4). The highest RUB is also the one with the fewest patients included; only 41, 54 and 71 patients are classified in RUB 5 through the different years (Table 5).

**Table 2:** Gender of inhabitants at public and private PHC.

	**2004 Proportion (%)**		**2005 Proportion (%)**		**2006 Proportion (%)**	
**Gender**	**Public**	**Private**	**Public**	**Private**	**Public**	**Private**

Female	49.84	50.58	49.96	49.05	50.07	47.92
Male	50.16	49.42	50.04	50.95	49.93	52.08

Total	100.00	100.00	100.00	100.00	100.00	100.00

PHC - primary health care

**Table 3:** Means of costs for specific RUB with confidence interval, 2004-2006

	**2004**			**2005**			**2006**		
			
**RUB**	**Mean**	**[95% Conf.** ** interval]**		**Mean**	**[95% Conf.** ** interval]**		**Mean**	**[95% Conf.** ** interval]**	
RUB 0	483.75	460.29	507.21	507.90	482.67	533.13	540.08	512.94	567.23
RUB 1	1850.11	1804.83	1895.38	1942.94	1889.78	1996.09	2532.25	2470.52	2593.98
RUB 2	2635.64	2569.7	2701.57	2737.82	2674.31	2801.33	3466.08	3402.96	3529.2
RUB 3	5642.28	5472.63	5811.93	5861.66	5677.63	6045.69	6811.42	6661.08	6961.76
RUB 4	10720.11	9571.21	11869.02	12378.67	10822.5	13934.84	12198.62	11119.78	13277.47
RUB 5	26034.18	14219.38	37848.99	16519.24	10906.28	22132.2	20083.55	15676.78	24490.32

(1 USD = 8.56 SEK; 22 April, 2009)

RUB - resource utilization band

**Table 4:** Degree of explanation of concurrent log-costs, model 1-5

	**2004**			**2005**			**2006**		
			
**Model**	**Adjust r^2^**	**F**	**P Value**	**Adjust r^2^**	**F**	**P Value**	**Adjust r^2^**	**F**	**P Value**
1	0.0167	2050.84	< 0.0001	0.0177	2263.55	< 0.0001	0.0187	2328.50	< 0.0001
2	0.1399	188.10	< 0.0001	0.1433	198.79	< 0.0001	0.1425	194.72	< 0.0001
3	0.1448	180.38	< 0.0001	0.1482	190.66	< 0.0001	0.1488	188.66	< 0.0001
4	0.6053	1556.98	< 0.0001	0.6107	1639.94	< 0.0001	0.6308	1758.16	< 0.0001
5	0.6089	1028.16	< 0.0001	0.6137	1101.62	< 0.0001	0.6341	1159.97	< 0.0001

Model 1: Gender

Model 2: Gender and age

Model 3: Gender, age and PHC

Model 4: Gender, age, PHC and RUB

Model 5: Gender, age, PHC and ACG

ACG - Adjusted Clinical Groups, PHC - primary health care site

**Table 5:** Number of inhabitants in each RUB by age group 2004-2006

**2004**							
**Age**	**RUB 0**	**RUB 1**	**RUB 2**	**RUB 3**	**RUB 4**	**RUB 5**	**Total**

0-19	19 083	4 201	4 282	450	3	0	28 019
20-39	20 071	3 864	4 409	1 395	8	0	29 747
40-59	18 570	3 657	5 916	3 072	64	1	31 280
60-79	10 525	2 232	5 361	5 425	250	18	23 811
80-	2 805	618	1 483	2 733	220	22	7 881

Total	71 054	14572	21 451	13 075	545	41	120 738

2005							

**Age**	**RUB 0**	**RUB 1**	**RUB 2**	**RUB 3**	**RUB 4**	**RUB 5**	**Total**

0-19	17 792	5 115	5 118	608	3	0	28 636
20-39	20 245	4 217	4 508	1 461	9	0	30 440
40-59	18 689	4 135	6 150	3 349	77	2	32 402
60-79	10 948	2 468	5 844	6 012	260	24	25 556
80-	2 787	742	1 686	2 863	232	28	8 338

Total	70 461	16 677	23 306	14 293	581	54	125 372

2006							

**Age**	**RUB 0**	**RUB 1**	**RUB 2**	**RUB 3**	**RUB 4**	**RUB 5**	**Total**

0-19	16 655	5 102	5 232	619	5	0	27 613
20-39	18 758	4 206	4 703	1 561	7	1	29 236
40-59	17 324	4 035	6 259	3 714	82	6	31 420
60-79	10 150	2 531	6 008	6 611	349	35	25 684
80-	2 579	757	1 674	3 109	292	29	8 440

Total	65 466	16 631	23 876	15 614	735	71	122 393

RUB - resource utilization band

The adjusted R^2 ^measure shows that the co-morbidity, as measured by ACG case-mix, contributes most to the explanation rate in all the models examined. The adjusted R^2 ^is calculated as at least 0.0167 in model 1 and rising when more explanatory variables are added to the concurrent models. The data for 2006 consistently generate the highest adjusted R^2 ^for all models, while the results for 2004 and 2005 are similar to each other and with lower coefficient of determination (Table 4).

## Discussion

The ACG case-mix system is more widely used in countries other than Sweden and has been shown to be a very useful tool for explaining individual health care resource consumption [8,16]. It has not hitherto been used for allocation of funds in Sweden. This article presents a study of how the system works in a Swedish PHC setting.

As in other countries, equity in health care is considered one of the fundaments, established by law in Swedish health care [17-19]. Sweden has one of the oldest populations in the world. With demographics as the only predictor of health care need when allocating health care resources in PHC, it is unlikely to fulfil the aim of equity in health care. Several PHC organizations in Sweden base - or have based - their capitation mainly upon the age of the inhabitants within the county council. In a Swedish setting, age and gender have been shown to explain about 11 percent of the variation in primary costs [11]. Similar findings have been obtained in Canadian settings (9 percent) [9]. In the Swedish example, which only included two different PHC practices, the figure increased to 38 percent when ACG was added. In the Canadian study where ADGs were examined, the result reached 53 percent. Our study presents a result of age and gender explaining about 14 percent of the variance in log-cost. By also adding co-morbidity as measured by RUB or ACG in concurrent models, the results range from 60.53 to 63.41 percent explanation of the variation of primary care log-cost. Unlike in an earlier Swedish study [11] we have used log-cost as the dependent variable because individual costs of PHC in a population are highly positively skewed. Transforming of variables with the logarithmic function enabled us to analyse data with the linear regression method which also explains why we found a higher degree of explained variation than in the earlier Swedish study. However we did not use a cut-off point to exclude patients with high cost which was done in the cited study which would improve the degree of explained variation. This is the first time in Sweden data from a whole county with users and non-users of PHC have been analysed. We have also used RUB groups and ACG groups in separate models and found that the ACG groups as an independent variable does not improve the model much.

As has been shown in this study, the ACG case-mix system when used in a Swedish setting has been able to explain much of the variance in log-cost in health care and thus provides one factor that can enable equitable health care. This might be of special importance to the groups of elderly where co-morbidity is more common [2]. By focusing only on single diagnoses instead of co-morbidity, there is a serious risk of the elderly not being given the access to the health care resources they need. There are possibilities to investigate differences by dividing the population into smaller geographic areas. As suggested, the variation in health care need may be greater in small geographic areas than between greater geographic areas [17,20]. There are thus implications for policymakers to strive for equity by introducing a system based on individual patient-level co-morbidity.

The different models examined in this article imply that systematic use of the ACG case-mix system can be the foundation for adequate resource allocation in Swedish PHC. However, the results of more adequate resource allocation are likely to be seen not solely in PHC, as there is evidence that a strong PHC contributes to lower costs of care, but also to improve the performance of the health care system [21,22]. Rates of referral to specialist care vary between different countries and higher rates are likely to be one of the reasons for increasing costs in care [23,24]. Although there is much yet to be explained, parts of the variation in referral rates within a system can be explained by morbidity [25]. This gives possibilities to better foresee the use of specialist care and the contribution to lower costs in care. Being aware of and controlling for co-morbidity also gives an opportunity to better treat complex patients, with the effect of reducing the number of hospitalizations [26].

As Swedish health care does not automatically calculate individual patient costs, calculations are made afterwards based mainly upon time consumption by all registered activities. These calculations are thus estimates of the true costs and create an extra uncertainty when estimating individual costs compared to the ACG classifications. The possibilities of such calculations are dependent on the registration of all activities performed, and on the data system for registration of the time worked by the personnel.

The ACG case-mix system is also dependent on registration, the diagnoses registered by PHC physicians. As the system is founded on the diagnoses of patients, the extent of registration in electronic patient records is important [6].

Registration in Sweden and Blekinge specifically, however, has been shown to be on a fairly good level. In a one-year retrospective study in Blekinge County Council about 45 percent of the inhabitants had at least one diagnosis-registered encounter with a GP in the year 2002 [10]. When a time period of three years was examined instead, about three out of four inhabitants had at least one diagnosis-registered encounter with a GP and the percentage of encounters with a diagnosis registered almost reached 90 percent [27]. Also, the similar results in this study over the three years somewhat proves the stability of the diagnosis registration during the study period.

At the same time, the dependency on registration is also one of the strengths of system, as it is based upon information already being registered, in combination with demographics [18]. All false or missing registration of data can be a possible source of bias in the results, unless it is random. Strategic manipulation is to some extent possible but it will be effective in a more complete documentation. For the entire organization or county council, the up-coding will result in a zero-sum game [1,16]. A capitation system based on age and gender is very unlikely to be strategically manipulated.

The last year examined in this study, 2006, has the highest coefficient of determination in the different models. Possible explanations are higher quality of diagnosis registration, better registration of all elements in patient cost calculations and more exact patient cost calculations. Still, co-morbidity is shown in the study to explain much of the calculated log-cost in the models presented. The calculations also seem stable over time, considering the results of the three years examined.

There are other factors that contribute to the results of the classification in the ACG case-mix system, such as the period of time chosen for examination. In this study the period corresponds to one year, but both shorter and longer time intervals have been used in different contexts. Only inhabitants enrolled with public PHC, corresponding to more than 80 percent for all years, were included in the study. The share of patients enrolled in private PHC is small enough to be considered not to influence the result in the study in a negative way. Co-morbidity has been shown to explain the variation of patient log-cost in PHC to a high degree, but factors outside these models may also have a great impact on the results. The concept of frequent attenders in primary care corresponds to the number of inhabitants in the population who use health care resources to a disproportionately high degree [28]. This small group of patients, about 3 percent, accounts for about 25 percent of the visits to primary care physicians [28]. The frequent attenders are also to a higher degree referred to hospital specialist than other patients [29]. At the same time, the presence of, for example, chronic disease among the frequent attenders is substantial and it is not possible to rule out that the visits are medically adequate.

Returning to the statement that the main objective of health care is to improve health, the ACG case-mix has shown to be an important part of such a mission that can help to allocate resources according to the need. The usefulness lies within creating a model for adequate resource allocation within a PHC organization. Such a foundation provides the basic possibilities for equitable primary health care. ACG case-mix is currently being introduced in Swedish PHC using concurrent reimbursement models. Aims for future research would be to develop and validate prospective funding using predictive models and how they would benefit from adding diagnoses from specialised health care. A prerequisite to develop such models which would work in practice is the ongoing work to improve diagnostic practices in Swedish PHC.

## Conclusion

The ACG case-mix system explains patient log-cost in PHC to a high degree. Age and gender are important explanatory factors, but most of the variance in patient log-cost is explained by the ACG case-mix system.

## Competing interests

The authors declare that they have no competing interests.

## Authors' contributions

AZ drafted the manuscript and participated in the design of the study. MK helped draft the manuscript. HL performed the statistical analysis and participated in the design of the study. AH helped in the statistical analysis and drafting the manuscript, handled the data set and designed the study. All authors read and approved the final manuscript.

## Pre-publication history

The pre-publication history for this paper can be accessed here:


